# Immersive Virtual Reality as Computer-Assisted Cognitive–Motor Dual-Task Training in Patients with Parkinson’s Disease

**DOI:** 10.3390/medicina61020248

**Published:** 2025-02-01

**Authors:** Lucie Honzíková, Marcela Dąbrowská, Irena Skřinařová, Kristýna Mullerová, Renáta Čecháčková, Eva Augste, Jana Trdá, Šárka Baníková, Michal Filip, David Školoudík, Iva Štefková, Vojtěch Štula

**Affiliations:** 1 Department of Rehabilitation and Sports Medicine, Faculty of Medicine, University of Ostrava, 70300 Ostrava, Czech Republic; michal.filip@osu.cz (M.F.); iva.stefkova.s01@osu.cz (I.Š.); vojtech.stula.s01@osu.cz (V.Š.); 2 Centre for Information Technology, Artificial Intelligence and Virtual Reality in Medicine, Faculty of Medicine, University of Ostrava, 70300 Ostrava, Czech Republic; 3 Department of Rehabilitation and Sports Medicine, University Hospital of Ostrava, 70852 Ostrava, Czech Republic; irena.skrinarova@fno.cz (I.S.); kristyna.pasterniakova@seznam.cz (K.M.); renata.cechackova@fno.cz (R.Č.); sarka.banikova@fno.cz (Š.B.); 4 Institute of Physiology and Pathophysiology, Faculty of Medicine, University of Ostrava, 70300 Ostrava, Czech Republic; eva.augste@osu.cz; 5 VR Vitalis, 70900 Ostrava, Czech Republic; jana.trda@vrvitalis.com; 6 Department of Clinical Neurosciences, Faculty of Medicine, University of Ostrava, 70300 Ostrava, Czech Republic; david.skoloudik@osu.cz

**Keywords:** balance, clinical trials, mobility, neurodegenerative diseases, questionnaire, rehabilitation

## Abstract

*Background and Objectives*: The aim of this study was to determine the effect of immersive virtual reality used as a short-term multifaceted activity with a focus on motor and cognitive function in patients with Parkinson’s Disease. The sub-objective focused on quality of life in the study group of patients. *Materials and Methods*: Nineteen patients (64.2 ± 12.8 years) were included in this study. Inclusion criteria for this study: adult patients in Hoehn and Yahr’s stage 1–3, cooperative, with stable health status, independent and mobile. IVR therapy was performed twice a week for 20 min for one month. Input and output measurements were taken within 14 days of starting or ending therapy. The 10 Meter Walk test was used to examine and assess both comfortable and fast walking, and the Timed Up and Go (TUG) + s dual task was applied to quickly assess the highest possible level of functional mobility. The Berg Balance Scale test (BBS) was used to assess balance with a 14-item balance scale containing specific movement tasks. The standardized Parkinson’s Disease Questionnaire (PDQ-39) was used to assess quality of life. Data were processed in the PAST program using a nonparametric paired Wilcoxon test. The significance level was set at α = 0.05. The value of the r score was used to evaluate the effect size. *Results*: A significant reduction in the time in the fast walk 10MWT (*p* = 0.006; r = 0.63) and TUG (*p* < 0.001; r = 0.80) parameter were found after therapy. Significant improvement in the BBS score was found after applied therapy (*p* = 0.016; r = 0.55). In the PDQ-39 questionnaire, significant improvements were found in the study group after therapy in the domains of mobility (*p* = 0.027; r = 0.51) and emotional well-being (*p* = 0.011; r = 0.58). *Conclusions*: The results of this study indicate a positive effect of virtual reality therapy on balance and gait, which is also good in terms of reducing the risk of falls in the study group. Therapy also promoted quality of life in the study group.

## 1. Introduction

Parkinson’s Disease (PD) is a disease characterized by typical motor manifestations that include impaired balance (postural instability), slowed movement (bradykinesia), resting tremor, and increased muscle rigidity, with a progressively worsening course. While pharmacological treatments for PD may provide relief from certain motor symptoms, managing mobility and postural control challenges remains a significant difficulty for patients as the disease progresses. Additionally, PD is associated with non-motor symptoms, such as cognitive impairments, which further complicate the daily lives of those affected [1]. Multitasking plays a key role in everyday life as it often requires the simultaneous performance of cognitive and motor tasks [2]. This ability allows individuals to perform complex activities such as moving through an environment coupled with the perception of potential obstacles and risks [3]. Coordination between cognitive functions and motor activities is an important factor for the efficient and safe movement in different situations. This phenomenon is referred to in the literature as cognitive–motor interference, cognitive training, or dual task [2,3,4]. Cognitive–motor interference requires the patient to perform motor and cognitive tasks simultaneously. This approach provides information about automation [2].

Individuals with Parkinson’s Disease often exhibit diminished abilities in performing cognitive–motor tasks compared to healthy individuals of similar age, sex, and educational background [4,5]. This highlights the relevance of therapeutic interventions targeting these areas. Cognitive–motor dual-task training (CMDT) focuses on improving both motor and cognitive functions simultaneously, thereby enhancing the overall efficacy of functional therapy and strengthening cognitive–motor coordination [6]. The dual-task approach involves executing two distinct goal-oriented activities concurrently, such as solving basic arithmetic problems while walking a specified distance [5]. Engaging motor and cognitive functions simultaneously in dual-task training has been shown to offer additional benefits compared to single-task training protocols [7].

Cognitive–motor dual-task training (CMDT) can be performed with or without the support of technology systems, although the integration of advanced technologies improves the monitoring of therapeutic progress, provides real-time feedback, and promotes greater patient engagement [8,9,10]. In recent years, there has been a notable expansion in research utilizing computer-assisted devices within interventions aimed at enhancing treatment efficacy and improving the accuracy of diagnostic evaluations. One such technology is immersive virtual reality (IVR), which facilitates authentic cognitive–motor challenges by simulating real-world interactions and offering multisensory experiences within a virtual environment [11,12]. When this technology enters therapy, we talk about computer-assisted cognitive–motor dual-task training (CACMDT) [13]. Long-term therapy is needed for patients with PD, and we wondered if IVR would be tolerated by patients and therefore may be an appropriate therapy choice. The aim of this study was to determine the effect of immersive virtual reality used as a short-term multifaceted activity with a focus on motor and cognitive function in patients with Parkinson’s Disease. The sub-objective focused on quality of life in the study group of patients.

## 2. Materials and Methods

### 2.1. Study Design

This study was carried out according to the principles of the Declaration of Helsinki and was approved by the Ethics Committee of the University Hospital in Ostrava (reference number 57/2024).

### 2.2. Participants

Participants were recruited through the outpatient clinic of the University Hospital in Ostrava. All participants provided their written informed consent.

A total of 19 patients (64.2 ± 12.8 years) participated in this study. Inclusion criteria for this study: adult patients in Hoehn and Yahr stages 1–3 [14], cooperative, with stable health, independent and mobile, without visual, hearing, speech or cognitive impairment, and without severe neurological disease (epilepsy, vertigo, etc.). The Addenbrooke’s cognitive examination (ACE III) was used to classify cognition, and the group of patients was within normal limits in the overall assessment (score 90). Only for visual–spatial ability were they borderline dementia (score 14) [15].

### 2.3. Procedures

The 10 Meter Walk test (10MWT) was used to examine and assess both comfortable and fast walking [16]. The Timed Up and Go test (TUG, standing and walking test) + dual task (DT) was applied to quickly assess the highest possible level of functional mobility [17]. To assess balance, the Berg Balance Scale (BBS) test, a 14-item balance scale that contains specific movement tasks, was used [18]. The standardized Parkinson’s Disease Questionnaire (PDQ-39) was used to assess quality of life, consisting of 39 questions, eight domains: mobility, daily life, emotions, stigma, communication, and physical discomfort [19].

### 2.4. Instruments Used

The Meta Quest 2 headset (Meta Platforms, New York, NY, USA) was used for the virtual reality application. The device includes 6 GB of RAM and the Qualcomm Snapdragon XR2 platform for high performance. The system is equipped with a projection headset featuring LCD displays, each offering a resolution of 1832 × 1920 pixels per eye, delivering a visual clarity of approximately 21 pixels per degree, ensuring an immersive experience without visual artifacts. The helmet is equipped with adjustable straps and is compatible with dioptric glasses. The two touch controls include control buttons, joystick, and anti-slip fixation straps. The glasses are connected via Bluetooth to a mobile app on a tablet that allows the therapist to monitor, control, and analyze the patient’s performance on tasks.

### 2.5. Intervention

The virtual reality software VITALIS Pro version 0.4.1 was used for the therapy, in which modules including exercises with dual task components were developed for Parkinson’s patients.

For the upper limbs, the modules were as follows:

- Hanging laundry (laundry)—range of motion support;
- Watering flowers (flowers)—focus on precision;
- Catching butterflies (butterflies)—developing range of motion;
- Opening doors with keys (keys)—training fine motor skills;
- Chopping wood (chopping)—focus on range of motion;
- Folding mugs (mugs)—focus on the shape and color of the mug.

For the lower limbs, the modules were as follows:

- Stomping in puddles (puddles)—agility of movement;
- Kicking the ball (kicking)—focus on control of movement;
- Walking on tracks without obstacles (steps)—education of walking;
- Walking on tracks with obstacles (steps—obstacles)—gait education and fall prevention.

Exercises were designed to maintain range, fluency, and the accuracy of movement and to promote patient reactivity. The individual tasks were conducted within three thematic environments, each enhanced by contextually relevant auditory stimuli: the forest, accompanied by birdsong; the seaside, with the sound of waves; and outer space, featuring computer-generated music, all of which contributed to the overall immersive experience.

In addition to a group conventional exercise of 30 min once a week, the group received rehabilitation in IVR 10–20 min twice a week according to the patient’s tolerance in the constant physical presence of a physiotherapist or occupational therapist.

Baseline and outcome tests were performed up to a maximum of 14 days before therapy and up to 14 days after therapy.

### 2.6. Statistical Methods

Statistical analysis of the data was performed in the PAST4.08c program [20]. The mean, standard deviation, median, and upper and lower quartile were calculated for each monitored variable. Due to the small number of probands, the non-parametric paired Wilcoxon test was used to evaluate the differences in the observed parameters between individual measurements within the given group. Differences at the level of statistical significance α = 0.05 were considered statistically significant. The value of the r score (r = Z/sqrt[N], where N is the total number of observations) was used to evaluate the effect size. The effect size was considered large for values ≥0.5, moderate for values ≥0.3 and <0.5, ≥0.1 and <0.3 small, and <0.1 trivial [21].

## 3. Results

Table 1 shows the values of the clinical gait and stability tests. After therapy, there were significantly lower times measured during the fast walk 10MWT and significantly lower times after therapy during the TUG test. The BBS test showed significant improvement in the score after the applied therapy.

Table 2 shows the scores for each domain of the PDQ-39 quality of life questionnaire, as well as the total score. Significant improvements were found in the domains of mobility and emotional well-being. The overall score characterizing quality of life showed improvement after therapy in the study group but was not statistically significant.

## 4. Discussion

Computer-assisted cognitive–motor dual-task training (CACMDT) integrates cognitive and motor tasks into activities requiring the coordinated activation of these functions. This approach is key in the neurorehabilitation of Parkinson’s Disease (PD), where it helps to improve coordination between motor and cognitive functions. CACMDT promotes neuroplasticity, thereby slowing symptom progression and improving quality of life for PD patients. Cognitive functions are closely related to motor abilities in PD. Therefore, in our study, we chose to use IVR therapy to promote cognitive–motor interference, as was performed in studies. The main benefit of this therapy is to increase motor automaticity with minimal conscious attention or executive control [1,13,22,23,24], but under the simultaneous supervision of a physiotherapist. Single-task training (ST) is often used in rehabilitation to improve specific motor skills and does not present the same cognitive load as CACMDT [25]. Although single-task training (ST) can result in improvements in specific motor functions, it may not effectively address the cognitive–motor interference frequently encountered during everyday activities. Some degree of cognitive impairment is manifested by a change in postural control prioritization, the loss of rhythmicity, and a reduction in gait speed. As a result, there is a high risk of falling [2]. In our study group of patients with PD, cognitive–motor therapy was specifically targeted at stability and gait optimization. Even though our patients were in the low fall-risk region of the BBS (according to the Berg et al. [18] study, this is a score range of 41–56), before therapy, PD patients had a score of 52.1, and we felt that an assessment of this factor after therapy would be important in terms of postural confidence because of therapy. After therapy in the IVR, we found significant improvements in the fast walk 10MWT test, reactivity in the TUG test and stability in the BBS score in our study group of patients with PD. The results emphasize the crucial role of integrating CACMDT into rehabilitation programs for patients with PD, as well as its inclusion in comprehensive fall prevention strategies. Improved stability had a positive impact on patients’ mobility, which was also reflected in the quality of life questionnaire. There was also a significant improvement in quality of life in a study [26] that focused on cognitive–motor therapy.

The preservation of walking ability is a primary concern for patients with PD. Physicians focus primarily on cadence, stride length, and gait speed. These parameters are crucial for measuring individual independence and are possible indicators of other problems, such as stability [27]. Moreover, gait parameters are closely correlated with the severity of Parkinson’s Disease, as assessed through various clinical scales. Alterations in these parameters reflect limitations in physical activity and an overall worsening of disability while also providing valuable insight into the response to treatment, making them essential for evaluating the effectiveness of interventions aimed at addressing gait dysfunction in PD. Furthermore, they are widely recognized as reliable predictors of an individual’s ability to walk independently within the community. In our study group of patients with PD, there was not change in walking speed in the TUG test with DT after VR treatment. The work of Raffegeau et al. [28] showed that the addition of a DT during walking had a moderate to large negative effect on walking speed in individuals with PD, regardless of the single-task or dual-task type. Dual tasks severely and meaningfully affect walking in people with Parkinson’s Disease, which was also evident in our TUG + DT test. However, in the TUG test without a DT, significant gait acceleration occurred in the PD patients that we studied, and, in the fast walk 10MWT test, there was significant decreased time after therapy. A study conducted by Tan et al. [13] reports that participants in the dual-task (DT) therapy group exhibited a statistically significant increase in walking speed following the intervention, with this improvement sustained throughout the follow-up period, even under various conditions. Gait acceleration indicates improved stability and motor control during normal and fast walking. Significant improvement mobility was also reported by follow-up patients in the PDQ39 quality of life questionnaire. This improvement also entailed significant improvements in emotional well-being, as self-reported by patients on the PDQ39 quality of life questionnaire. Cognitive functions, particularly executive function and attention, are key determinants of mobility. Consequently, rehabilitation approaches that address both cognitive and motor deficits are likely to yield substantial benefits for improving mobility outcomes [15,17,29].

### Limits

A major limitation of this study is the small sample size of patients with PD. This is due to the low motivation to participate in this study due to PD-related discomfort. For example, medication is altered in PD, which carries a negative impact related to adaptation to medication, mood changes, impaired concentration, rapid fatigue, the possible occurrence of increased sweating. And also changes in weather affecting the patient’s perception and mood.

## 5. Conclusions

The study cohort of patients with Parkinson’s Disease engaged in immersive virtual reality (IVR) therapy. Cognitive–motor dual-task training within the IVR (CACMDT) emerges as a promising neurorehabilitation approach for individuals with Parkinson’s Disease, combining a dual focus on enhancing motor automaticity and cognitive function. The patient group demonstrated a marked improvement in stability, mobility, and overall quality of life. IVR-based therapy provides a comprehensive and adaptable strategy to address the complex challenges associated with Parkinson’s Disease.

## Figures and Tables

**Table 1:** Median, lower, and upper quartile values and results of statistical comparison of measurements for clinical test values.

Monitored Parameter	M_1_ (Q1; Q2)	M_2_ (Q1; Q2)	*p*	r
10 MWT comfortable [s]	9.0 (8.5; 9.8)	9.2 (7.8; 10.3)	0.409	0.19
10MWT fast [s]	6.6 (6.0; 8.0)	6.3 (5.8; 7.9)	**0.006**	**0.63**
TUG [s]	9.2 (8.8; 12.7)	8.7 (8.1; 10.7)	**<0.001**	0.80
TUG+DT [s]	11.2 (9.8; 15.3)	10.2 (8.7; 14.1)	0.243	0.27
BBS [score]	54.0 (52.0; 56.0)	56.0 (54.0; 56.0)	**0.016**	**0.55**

Explanatory notes: M_1,2_—median before therapy (1) and after therapy (2); Q1; Q2—quartiles lower (1) and upper (2); 10MWT—10 Meter Walk test; TUG—Timed Up and Go test; TUG+DT—Timed Up and Go test+dual task; BBS—Berg Balance Scale test; *p*—probability value; and r—effect size coefficient.

**Table 2:** Median, lower, and upper quartile values and results of statistical comparison of measurements for scores of individual domains of the PDQ-39 quality of life questionnaire.

PDQ-39 Scales	M_1_ (Q1; Q2)	M_2_ (Q1; Q2)	*p*	r
Mobility	30.0 (12.5; 37.5)	17.5 (10.0; 27.5)	**0.027**	**0.51**
ADLs	29.2 (8.3; 45.8)	16.7 (8.3; 37.5)	0.053	0.44
Emotional Well-Being	20.8 (8.3; 29.2)	12.5 (4.17; 29.2)	**0.011**	**0.58**
Stigma	6.3 (0.0; 31.3)	6.3 (0.0; 25.0)	0.304	0.24
Social support	8.3 (0.0; 25.0)	0.0 (0.0; 25.0)	0.160	0,32
Cognition	18.8 (6.3; 37.5)	18.8 (12.5; 37.5)	0.477	0.16
Communication	16.7 (0.0; 25.0)	16.7 (0.0; 33.3)	0.959	0.01
Bodily discomfort	25.0 (16.7; 41.7)	41.7 (16.7; 41.7)	0.329	0.22
PDSI	4.6 (2.3; 6.1)	3.2 (2.0; 6.4)	0.051	0.45

Explanatory notes: M_1,2_—median before therapy (1) and after therapy (2); Q1; Q2—quartiles lower (1) and upper (2); ADLs—activities of daily living; PDSI—summary index of PD (the lower the better the quality of life); M—arithmetic mean; SD—standard deviation; *p*—probability value; and r—effect size coefficient.

## Data Availability

All research data were securely stored in digital format and protected from loss and unauthorized access. The data presented in this study are available upon request from the corresponding author due to privacy reasons.

## Abbreviations

The following abbreviations are used in this manuscript:

10MWT	10 Meter Walk Test
ACE III	Addenbrooke’s Cognitive Examination
ADLs	Activities of daily living
BBS	Berg Balance Scale
CACMDT	Computer-assisted cognitive–motor dual task
CMDT	Cognitive–motor dual task
DT	Dual task
IVR	Immersive virtual reality
M	Arithmetic mean
p	Probability value
PD	Parkinson’s Disease
PDQ	Parkinson’s Disease Questionnaire
PDSI	Summary Index of Parkinson’s Disease
SD	Standard deviation
ST	Single Task
TUG	Timed Up and Go
